# Effects of chemical exposures and diet on birth outcomes in a New York City pregnancy cohort: Mediation through favorable fetal growth conditions

**DOI:** 10.1371/journal.pone.0322399

**Published:** 2025-05-28

**Authors:** Eunsil Seok, Akhgar Ghassabian, Yuyan Wang, Yelena Afanasyeva, Shilpi S. Mehta-Lee, Kurunthachalam Kannan, Leonardo Trasande, Mengling Liu

**Affiliations:** 1 Department of Population Health, New York University Grossman School of Medicine, New York, New York, United States of America; 2 Department of Pediatrics, New York University Grossman School of Medicine, New York, New York, United States of America; 3 Department of Obstetrics and Gynecology, New York University Grossman School of Medicine, New York, New York, United States of America; 4 Wadsworth Center, New York State Department of Health, Albany, New York, United States of America; Center for Research and Technology Transfer, VIET NAM

## Abstract

**Background:**

Fetal growth is shaped by a complex interplay of parental traits, environmental exposures, nutritional intake, and genetic predispositions. In epidemiological research, birth weight is widely used as a proxy of impaired or favorable fetal growth; but it fails to provide a comprehensive measure, particularly if used alone.

**Methods:**

In a cohort of 538 mother-fetal pairs from the New York University Children’s Health and Environment Study (NYU CHES), we utilized multiple linear regression and structural equation modeling (SEM) to assess the influence of various determinants–maternal characteristics, chemical exposures, and dietary factors–on fetal growth. To comprehensively evaluate fetal growth, we employed the concept of latent variable Favorable Fetal Growth Conditions (FFGC), together with three observed outcomes: birth weight, birth length, and gestational age.

**Results:**

Maternal characteristics such as height, BMI, race/ethnicity, and maternal alcohol intake were significantly associated with birth weight, birth length, and gestational age in both the linear regression and with FFGC in the SEM. However, SEM additionally revealed significant relationships that were not detected by linear regression. Specifically, di(2-ethylhexyl) phthalate (*DEHP*) latent factor showed a negative association with the FFGC (β=-0.16, 95% confidence interval (CI)=-0.27, -0.04). The diet latent variable positively impacted FFGC (β=0.15, 95% CI=0.04, 0.25), whereas total calorie intake exhibited a negative effect (β=-0.13, 95% CI=-0.22, -0.05).

**Conclusion:**

The SEM provided a thorough understanding of the multifaceted pathways through which multiple factors of chemical mixtures, diet intakes, and maternal characteristics affected fetal development, uncovering nuanced associations that were not apparent in direct effects models. Our findings highlight the intricate interplay of maternal characteristics, chemical exposures, and dietary factors in shaping fetal growth.

## 1. Introduction

Fetal growth represents a complex interplay of parental traits, environmental exposures, nutritional intake, and genetic predispositions, shaping the developmental trajectory of the fetus from conception to birth [1]. Research has highlighted the profound impact of adverse nutritional and environmental exposures during fetal development, with implications extending into adulthood, including increased susceptibility to metabolic syndrome, diabetes, hypertension, and cardiovascular disease [2–5]. Additionally, factors such as inadequate dietary intake, substance use, poverty, trauma, and high stress levels during pregnancy have been shown to directly or indirectly influence fetal growth conditions, emphasizing the intricate nature of this process [6].

Maternal characteristics are essential to fetal development and subsequent birth outcomes. Advanced maternal age, higher maternal body mass index (BMI), and maternal lifestyle such as smoking and alcohol consumption are associated with gestational diabetes and hypertension, leading to adverse birth outcomes, such as low birth weight and preterm birth [7–12]. Additionally, socioeconomic factors including income, education, and access to healthcare services play crucial roles, with disparities in these areas contributing to inequalities in maternal and infant health [13,14]. Maternal mental health, e.g., depression, has been associated with detrimental effects on fetal development [15,16]. Additionally, exposure to environmental chemicals, such as phthalates, bisphenols, and organophosphate (OP) pesticides during pregnancy poses a significant threat to fetal growth and development. Studies suggest that these exposures have been associated with adverse effects on neurobehavioral, reproductive, and hormonal development in children, operating via various mechanisms, such as endocrine disruption, epigenetic modifications, oxidative stress, and inflammation [17–21]. Furthermore, maternal diet is a critical determinant in both short-term and long-term health outcomes, where inadequate or excessive nutrient intake during pregnancy can lead to various negative birth outcomes, including fetal growth restriction, low or high birth weight, and increased susceptibility to obesity and chronic disease in adulthood [22–24].

While infant birth weight has been commonly used in epidemiological studies to characterize impaired or favorable fetal development and growth, it fails to provide a comprehensive measure of fetal growth, particularly if used alone [25]. Additionally, the conventional belief that heavier or taller fetuses equate to better outcomes is challenged; birth weight and length do not inherently signify a more favorable birth outcome. Favorable Fetal Growth Conditions (FFGC), a latent variable that encompasses key indicators such as birth weight, birth length, and gestational age, offers a comprehensive assessment of fetal growth, potentially addressing the limitation of using only birth weight [26]. The FFGC framework suggests that fetal growth is influenced by a complex interplay of genetic and environmental factors, with effects that may be either beneficial or detrimental throughout the lifespan. Previous research has examined the relationship between FFGC and various maternal characteristics, environmental exposures, and birth outcomes, underscoring its significance in understanding fetal development and subsequent health outcomes [26–30].

Structural equation modeling (SEM) [31,32] is a linear model framework that examines the relationship between measured variables and latent variables, and the relationship between latent variables. By accommodating measurement errors inherent in observed variables, SEM yields more precise estimations of theoretical constructs [33]. This methodological approach not only enables a comprehensive examination of complicated relationships but also unveils latent dynamics within datasets. Through the integration of multiple observed and unobserved factors, SEM facilitates a deeper understanding of the underlying mechanisms, ultimately contributing to the generation of more robust and nuanced research findings.

In this paper, we employ both traditional linear models and SEM to investigate the relationships between maternal characteristics, chemical exposures, and dietary factors with birth outcomes, focusing on the role of FFGC. Our approach aims to establish a framework that utilizes SEM and the latent construct of FFGC to better capture the underlying contributors to fetal growth. By modeling FFGC as a latent factor shaped by various maternal and environmental characteristics, we provide a more comprehensive analysis of how these factors collaboratively shape fetal development. Our primary objective is to assess the effects of these determinants on three birth outcomes as well as on FFGC, to deepen our understanding of the complex drivers of fetal growth and development.

## 2. Methods

### 2.1. Study population

The study population consisted of the participants enrolled in the New York University Children’s Health and Environment Study (NYU CHES), a prospective birth cohort in New York City, which was designed to identify environmental influences on child health outcomes [34]. Briefly, beginning in March 2016, NYU CHES actively recruited pregnant individuals attending three NYU-affiliated hospitals. Eligible participants were those aged ≥18 years, within the first 18 weeks of pregnancy, spoke English, Spanish, or Chinese, and were planning to deliver at three NYU Langone Hospitals. Between March 22, 2016 and April 13, 2023, a cohort of 4,439 pregnant individuals enrolled in NYU CHES, and the data were accessed on December 19, 2023. Among those who provided biospecimens (up to three time points in pregnancy), 994 participants were selected for chemical measurements. Within this subset, 547 participants completed detailed dietary assessments and mental health questionnaires at the early, mid, and late stages of pregnancy. Following the exclusion of one extremely preterm birth (GA=26), four cases of multiple pregnancies (twins), and four participants with missing covariate data, the final sample for the analysis consisted of 538 mother-fetal pairs (S1 Fig). Overall characteristics of participants included in the analysis were not different from those excluded due to missing exposure and/or outcome data (S2 Table).

All participants provided written informed consent and the study was approved by the Institutional Review Board of the New York University Grossman School of Medicine.

### 2.2. Determinants of fetal growth

#### 2.2.1. Maternal demographic information.

Information on sociodemographic characteristics including race and ethnicity, educational level, employment status, and lifestyle factors, such as lifetime alcohol intake were collected through repeated questionnaires during prenatal visits and at birth. Data on maternal age at enrollment, parity, and infant sex were retrieved from electronic health records. Pre-pregnancy weight and height were extracted from electronic health records or self-reported if missing, and were used to calculate pre-pregnancy BMI. Urinary cotinine levels across pregnancy were used to assess tobacco exposure either active or passive smoking during pregnancy. Pregnant individuals who had urinary cotinine levels over the limit of detection (LOD, 0.013 ng/mL) were defined as having tobacco exposure during pregnancy.

#### 2.2.2. Measurement of chemicals.

Single random spot urine samples were collected at clinical care visits in early pregnancy (<18 gestational weeks), mid-pregnancy (18–25 gestational weeks), and late pregnancy (>25 gestational weeks). Participants were expected to provide urine samples at three time points, and the majority did. However, some participants provided only one (5.58%) or two (16.91%) samples. Samples were collected in polyethylene containers and aliquoted into bisphenol- and phthalate-free tubes, then stored at -80°C until chemicals were analyzed for three classes: bisphenols (BPs), phthalates, and OP pesticides.

Eight BPs were analyzed using liquid-liquid extraction followed by high-performance liquid chromatography electrospray ionization tandem mass spectrometry (HPLC-MS/MS) at the NYU Human and Environmental Exposure Analysis Laboratory. Twenty two phthalate metabolites were characterized using enzymatic deconjugation of glucuronidated phthalate monoesters followed by solid phase extraction coupled with HPLC-MS/MS. Moreover, six dialkyl phosphate (DAP) metabolites of OP pesticides were analyzed using HPLC-MS/MS. Among them, chemical concentrations with 60% or more of the sample concentrations below the LOD were excluded from the further analysis. As a result, 2 BPs, 9 phthalate metabolites, and 5 DAP were included in the analysis. Percentiles below the LOD, median, and interquartile range (IQR) of included metabolites are presented in S3 Table, and the correlation plot is in S4 Fig.

For statistical analysis, samples falling below the LOD, chemical concentrations were substituted with a single value calculated as LOD/$\sqrt{}2$, where LOD is specific to each chemical. Urine dilution adjustment was conducted using creatinine concentration following the method described in Boeniger et al. [35]. To be specific, we scaled the observed chemical concentrations by the ratio of the batch- and time-specific median creatinine concentration to the observed creatinine concentration. Subsequently, we computed the pregnancy average of the adjusted chemical measurements across time points and applied a log-transformation due to their skewed distributions.

#### 2.2.3. Dietary assessment.

The electronic version of the Diet History Questionnaire II (DHQ-II) was used to assess participant’s dietary intakes during the previous year, including the pregnancy period [36]. The DHQ-II consists of 124 commonly consumed food items and includes frequency, portion size, and dietary supplement questions. We summed these questions to create 10 food groups as in Liu et al. [37]: Vegetables, Fruit, Grains, Dairy, Meat, Seafood, Egg, Nuts and seeds, Cooked dried beans and peas, and Soy products. Values of implausible daily energy intakes, less than 500 kcal or more than 6000 kcal, were excluded from analyses (n = 62) [38–41]. The log-transformed food group values are used in the analysis to reduce the skewness. Furthermore, total calorie intake was log-transformed as well in the model.

#### 2.2.4. Mental health questionnaire.

Maternal depressive symptoms were measured via Patient Health Questionnaire 9 (PHQ-9, total points=27) in each trimester. For analysis, the total score was averaged if measured multiple times and scores greater than or equal to 10 indicate depression [42].

### 2.3. Birth outcomes

We included three birth outcomes analyzed in this study, i.e., birth weight in kilograms (kg), birth length in centimeters (cm), and gestational age at birth in weeks. Information on birth weight and birth length was obtained from electronic health records, and if missing, by maternal report. All three birth outcomes were retained as continuous variables. Infant sex was included as a confounder, with male infants defining the reference group.

### 2.4. Statistical analysis

In our statistical analysis, maternal age was grouped into three categories: Younger (<20 years old), Middle (20–35 years old), and Older (>35 years old), with the middle group serving as the reference to allow for the exploration of non-linear effects. Race/ethnicity groups were combined into Hispanic vs. Non-Hispanic (Non-Hispanic White, Non-Hispanic Black, Asian, and Multiracial/Other). Education levels were categorized into four groups: Low (High school or less), Middle (Some college/Associate degree), Intermediate (Bachelor’s degree), and High (Post-graduate degree), with the high category as the reference group. Maternal employment (yes/no), alcohol intake (yes/no), and parity (yes/no) were treated as categorical variables, while maternal height and pre-pregnancy BMI were included as continuous variables in the models.

#### 2.4.1. Direct effects model.

Multiple linear regression models were used to directly investigate the effects of the mother’s characteristics, chemical exposures, and diet variables on three birth outcomes, birth weight, birth length, and gestational age, respectively.

#### 2.4.2. FFGC latent variable model.

**Fig 1:**
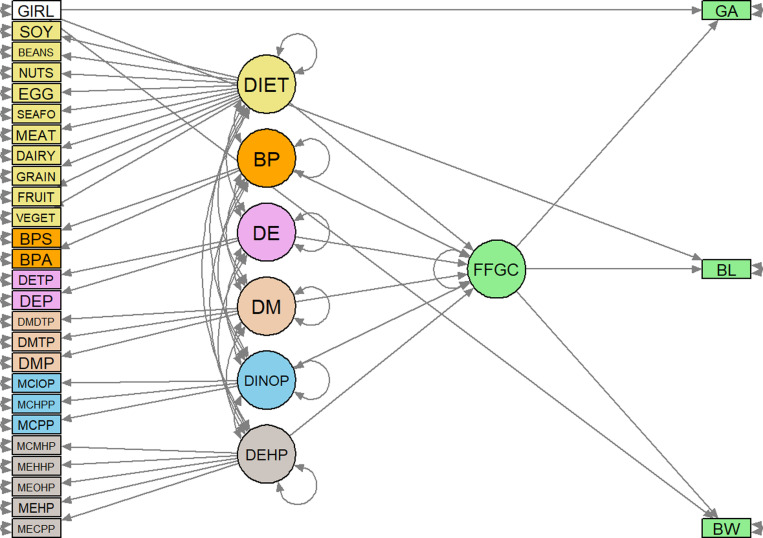
Directed acyclic graph (DAG) of FFGC latent variable model. Observed variables: GIRL = Infant is a girl; SOY = Soy products; BEANS = Beans & peas; NUTS = Nuts & seed; EGG = egg; SEAFO = Seafood; MEAT = Meat; DAIRY = Dairy; GRAIN = Grains; FRUIT = Fruit; VEGET = Vegetables; BPS = Bisphenol S; BPA = Bisphenol A; DETP = Diethylthiophosphate; DEP = Diethylphosphate; DMDTP = Dimethyldithiophosphate; DMTP = Dimethylthiophosphate; DMP = Dimethylphosphate; MCIOP = Mono-(carboxyisooctyl) phthalate; MCHPP = Mono-(7-carboxyheptyl) phthalate; MCPP = Mono-(3-carboxypropyl) phthalate; MCMHP = Mono-(7-carboxyheptyl) phthalate; MEHHP = Mono-(2-ethyl-5-hydroxyhexyl) phthalate; MEOHP = Mono-(2-ethyl-5-oxohexyl) phthalate; MEHP = Mono-(2-ethylhexyl) phthalate; MECCP = Mono-(2-ethyl-5-carboxypentyl) phthalate; GA: Gestational age; BL: Birth length; BW: Birth weight; Latent variables: DIET = diet; BP = Bisphenols; DE = Diethyl phosphate metabolites; DM = Dimethyl phosphate metabolites; DINOP = Diisononyl phthalate and di-n-octyl phthalate; DEHP = Di-(2-ethylhexyl) phthalate; FFGC = Favorable Fetal Growth Conditions.

Our SEM model contained a latent outcome variable, FFGC, based on the manifest variables of birth outcomes, as adopted from Bollen et al. [26]. In addition, our SEM incorporated six exogenous latent exposure variables: five chemical exposure variables–DEHP, DINOP, DM, DE, and BP–and one diet latent variable. For chemical exposure latent variables, we conducted exploratory factor analysis (EFA) to explore the structure of latent chemical exposure variables [43,44]. Additionally, ten food groups serve as indicators of diet latent variable, adjusted for total calorie intake. Fig 1 shows the directed acyclic graph (DAG) of the model elucidating how covariates, chemical exposures, and diet affect the latent outcome variable, FFGC, while accommodating a direct path from infant sex to the three birth outcomes, all of which are indicators of FFGC. Note that covariates other than infant sex were omitted in the DAG figure for ease of visualization.

To evaluate the overall fit of the SEM model, we employed several statistical indicators: Chi-square test statistic, Root Mean Square Error of Approximation (RMSEA), Standardized Root Mean Square Residual (SRMR), and Comparative Fit Index (CFI). The Chi-square test statistic assesses the overall fit by measuring the discrepancy between the sample and fitted covariance matrices. A p-value greater than 0.05 indicates that the hypothesis of a perfect fit cannot be rejected, though it is quite sensitive to sample size. The closer values of (1-RMSEA), (1-SRMR), and CFI are to 1, the better the model fits. Specifically, a cutoff criterion of at least 0.92 for (1-RMSEA) and (1-SRMR) is commonly used for defining a good fit [45,46], while a CFI value exceeding 0.90 is considered indicative of a well-fitting model [46,47].

All statistical analyses were performed using statistical software R (V4.3.3).

## 3. Results

### 3.1. Descriptive analysis

On average, participants were 31.72 years old (standard deviation (SD) = 5.53), with an average pre-pregnancy BMI of 25.95 kg/m^2^ (SD=5.52), and an average height of 160.82 cm (SD=8.57). Approximately half of the study population were nulliparous (51.12%), Hispanic (47.48%), and more than half held a college or higher degree (68.96%), were employed (68.77%), were not exposed to tobacco (67.47%), and had consumed alcohol at some point in their lives (65.61%). Moreover, among the 538 participants, 29 (5.39%) were categorized as experiencing depression. The overall mean birth weight and birth length of babies were 3.33 kg (SD=0.49) and 50.69 cm (SD=2.48), respectively. The median gestational age was 39.43 weeks (IQR=1.71). Table 1 summarizes the characteristics of the study participants.

**Table 1:** Summary of characteristics of study participants included in the CHES dataset.

Dataset	Variable	n=538
Birth outcome	Gestational age (weeks), median (IQR)	39.43 (1.71)
	Birth weight (kg), mean (SD)	3.33 (0.49)
	Birth length (cm), mean (SD)	50.69 (2.48)
	Infant sex, n (%)	
	Girl	267 (49.63)
Mother’s demographic information	Age (years), mean (SD)	31.72 (5.53)
	Height (cm), mean (SD)	160.82 (8.57)
	BMI (kg/m^2^), mean (SD)	25.95 (5.52)
	Parity, n (%)	
	Nulliparous	275 (51.12)
	Race/ethnicity, n (%)	
	Hispanic	256 (47.58)
	Non-Hispanic White	185 (34.39)
	Non-Hispanic Black	28 (5.20)
	Asian	50 (9.30)
	Multiracial/Other	19 (3.53)
	Education, n (%)	
	High school or less	167 (31.04)
	Some college	59 (10.97)
	Associate degree	30 (5.58)
	Bachelor’s degree	125 (23.23)
	Post-graduate degree	157 (29.18)
	Employment status, n (%)	
	Yes	370 (68.77)
	Tobacco exposure^a^, n (%)	
	Yes	175 (32.53)
	Alcohol intake (ever), n (%)	
	Yes	353 (65.61)
Diet^b^	Vegetables^c^, median (IQR)	1.79 (1.61)
	Fruit^c^, median (IQR)	1.74 (1.79)
	Grains^d^, median (IQR)	3.73 (2.83)
	Dairy^c^, median (IQR)	1.26 (1.21)
	Meat^d^, median (IQR)	3.39(3.94)
	Seafood^d^, median (IQR)	0.27(0.56)
	Egg^d^, median (IQR)	0.46 (0.78)
	Nuts & seeds^d^, median (IQR)	0.36 (0.98)
	Beans & peas^c^, median (IQR)	0.07 (0.16)
	Soy products^d^, median (IQR)	0.02 (0.05)
	Total calories, mean (SD)	1715.13 (890.76)
Depression^e^	Depression, n (%)	
	Yes	29 (5.39)

^a^Urinary cotinine levels were used to assess tobacco exposure. Levels above the LOD were categorized as ‘Yes,’ indicating detectable exposure, while levels below the LOD were categorized as ‘No.’; ^b^DHQ-II; ^c^Cup equivalents; ^d^Ounce equivalents; ^e^PHQ9.

### 3.2. Direct effects model

In the direct effects models, both maternal height and alcohol consumption significantly impacted all three birth outcomes, birth weight, birth length, and gestational age. Additionally, maternal BMI was observed to have a significant effect on birth weight (β=0.01, 95% CI=0.00, 0.02). Hispanics had significantly taller (β=0.71, 95% CI=0.11, 1.31) infants compared to other race/ethnicity. Moreover, female Infants were notably smaller in birth weight (β=-0.18, 95% CI=-0.26, -0.10) and shorter in birth length (β=-0.95, 95% CI=-0.95, -1.35) compared to male infants. Regarding gestational age as the outcome, older mothers tended to have shorter gestational age (β=-0.45, 95% CI=-0.75, -0.14), while nulliparous mothers had longer gestational age on average (β=0.41, 95% CI=0.12, 0.71). In terms of the chemical exposures, only two OP pesticide metabolites, namely, DEP and DMDTP, had significant negative effects on birth weight and birth length with coefficients of -0.07 (95% CI=-0.13, -0.01) and -0.16 (95% CI=-0.32, -0.01), respectively. None of the nutrition intakes showed any statistically significant impacts on birth outcomes. Further details are presented in Table 2.

**Table 2:** Beta coefficients and 95% confidence intervals from direct effect models.

	Birth weight (kg)		Birth length (cm)		Gestational age (weeks)	
	Estimate	95% CI^a^	Estimate	95% CI^a^	Estimate	95% CI^a^
Age group						
Younger	0.04	(-0.27, 0.34)	-0.31	(-1.82, 1.20)	-0.23	(-1.20, 0.75)
Middle	Ref	–	Ref	–	Ref	–
Older	-0.07	(-0.16, 0.03)	-0.44	(-0.91, 0.03)	-0.45	(-0.75, -0.14)*
Height (cm)	0.01	(0.01, 0.02)*	0.07	(0.04, 0.09)*	0.02	(0.00, 0.04)*
BMI (kg/m^2^)	0.01	(0.00, 0.02)*	0.04	(0.00, 0.08)	0.00	(-0.03, 0.03)
First pregnancy	-0.03	(-0.12, 0.06)	0.26	(-0.20, 0.71)	0.41	(0.12, 0.71)*
Tobacco exposure: Yes	0.01	(-0.07, 0.10)	0.08	(-0.35, 0.52)	-0.01	(-0.30, 0.27)
Alcohol exposure: Yes	0.18	(0.08, 0.28)*	0.68	(0.21, 1.16)*	0.51	(0.20, 0.82)*
Hispanic	0.11	(-0.01, 0.23)	0.71	(0.11, 1.31)*	0.36	(-0.03, 0.75)
Education						
Low	0.02	(-0.14, 0.19)	-0.30	(-1.12, 0.51)	0.02	(-0.51, 0.55)
Intermediate	0.03	(-0.12, 0.18)	-0.27	(-1.01, 0.48)	-0.22	(-0.71, 0.27)
Middle	0.02	(-0.10, 0.13)	0.17	(-0.39, 0.73)	-0.17	(-0.53, 0.20)
High	Ref	–	Ref	–	Ref	–
Unemployed	-0.01	(-0.11, 0.08)	0.16	(-0.32, 0.63)	0.05	(-0.26, 0.36)
Depression	-0.02	(-0.20, 0.17)	-0.38	(-1.29, 0.53)	-0.09	(-0.68, 0.51)
Girl infant	-0.18	(-0.26, -0.10)*	-0.95	(-0.95, -1.35)*	-0.00	(-0.26, 0.25)
DEHP						
mECPP	-0.13	(-0.27, 0.01)	-0.66	(-1.35, 0.03)	-0.09	(-0.54, 0.36)
mEHP	0.00	(-0.04, 0.05)	0.10	(-0.15, 0.34)	0.02	(-0.14, 0.18)
mEOHP	0.02	(-0.15, 0.19)	-0.27	(-1.13, 0.60)	-0.27	(-0.83, 0.29)
mEHHP	0.03	(-0.13, 0.20)	0.50	(-0.32, 1.32)	0.15	(-0.38, 0.68)
mCMHP	0.01	(-0.05, 0.06)	0.02	(-0.26, 0.30)	-0.05	(-0.23, 0.13)
DINOP						
mCPP	-0.00	(-0.06, 0.06)	-0.11	(-0.40, 0.19)	0.14	(-0.05, 0.33)
mCHpP	0.01	(-0.02, 0.04)	0.13	(-0.02, 0.27)	0.00	(-0.09, 0.10)
mCiOP	-0.00	(-0.05, 0.05)	0.07	(-0.19, 0.33)	-0.08	(-0.25, 0.09)
DM						
DMP	0.01	(-0.05, 0.07)	-0.04	(-0.35, 0.26)	0.08	(-0.12, 0.28)
DMTP	0.01	(-0.05, 0.06)	0.22	(-0.06, 0.49)	0.06	(-0.12, 0.24)
DMDTP	-0.02	(-0.05, 0.01)	-0.16	(-0.32, -0.01)*	-0.03	(-0.14, 0.07)
DE						
DEP	-0.07	(-0.13, -0.01)*	-0.25	(-0.56, 0.06)	-0.14	(-0.34, 0.06)
DETP	0.04	(-0.01, 0.08)	0.09	(-0.13, 0.31)	0.10	(-0.04, 0.24)
BP						
BPA	0.04	(-0.01, 0.08)	0.11	(-0.12, 0.33)	0.06	(-0.09, 0.21)
BPS	0.02	(-0.02, 0.06)	0.18	(-0.01, 0.38)	0.05	(-0.08, 0.18)
Diet						
Vegetables	0.04	(-0.04, 0.11)	0.09	(-0.27, 0.45)	0.12	(-0.11, 0.36)
Fruit	0.05	(-0.01, 0.12)	0.21	(-0.12, 0.53)	0.13	(-0.08, 0.34)
Grains	-0.02	(-0.13, 0.09)	0.02	(-0.54, 0.59)	0.09	(-0.27, 0.46)
Dairy	-0.02	(-0.09, 0.05)	0.02	(-0.33, 0.36)	0.04	(-0.19, 0.26)
Meat	-0.00	(-0.06, 0.06)	-0.11	(-0.42, 0.20)	-0.05	(-0.25, 0.15)
Seafood	0.02	(-0.02, 0.05)	0.07	(-0.10, 0.24)	0.03	(-0.07, 0.14)
Egg	0.01	(-0.04, 0.04)	-0.02	(-0.21, 0.18)	0.02	(-0.11, 0.15)
Nuts & seed	0.01	(-0.02, 0.05)	-0.02	(-0.17, 0.13)	0.03	(-0.07, 0.13)
Beans & peas	0.01	(-0.03, 0.05)	0.02	(-0.16, 0.20)	0.03	(-0.09, 0.15)
Soy products	-0.00	(-0.03, 0.03)	0.00	(-0.16, 0.16)	0.04	(-0.06, 0.15)
Total calorie	-0.11	(-0.32, 0.11)	-0.35	(-1.42, 0.73)	-0.62	(-1.32, 0.08)

^**a**^CI: Confidence interval; *Significant at the significance level α=0.05.

### 3.3. FFGC latent variable model

The results of fitting the model illustrated in the DAG shown in Fig 1 are provided in Table 3. All reported coefficients were standardized coefficients (std.all), enabling direct comparisons between observed and latent variables regarding their impacts on the outcome variable, given the uniform scaling applied to all variables.

**Table 3:** Beta coefficients and 95% confidence intervals from FFGC latent variable model.

	Estimate	95% CI^a^
Age group		
Younger	-0.01	(-0.10, 0.08)
Middle	Ref	–
Older	-0.07	(-0.16, 0.02)
Height	0.23	(0.13, 0.33)*
BMI	0.12	(0.03, 0.21)*
First pregnancy	0.04	(-0.06, 0.14)
Tobacco exposure: Yes	-0.00	(-0.09, 0.08)
Alcohol exposure: Yes	0.18	(0.08, 0.27)*
Hispanic	0.14	(0.02, 0.26)*
Education		
Low	-0.00	(-0.15, 0.15)
Intermediate	-0.01	(-0.12, 0.11)
Middle	0.01	(-0.09, 0.11)
High	Ref	–
Unemployed	0.01	(-0.09, 0.10)
Depression	-0.04	(-0.13, 0.05)
DEHP	-0.16	(-0.27, -0.04)*
DINOP	-0.03	(-0.19, 0.13)
DM	0.03	(-0.09, 0.14)
DE	-0.07	(-0.25, 0.11)
BP	0.22	(-0.05, 0.48)
Diet	0.15	(0.04, 0.25)*
Total calorie	-0.13	(-0.22, -0.05)*

^**a**^CI: Confidence interval; *Significant at the significance level α=0.05.

The Chi-square test statistic indicates a significance in the overall model fit, with a p-value <0.001, although this level of significance might be undesirable. Nonetheless, both the goodness of fit measures (1-RMSEA) and (1-SRMR) were at 0.928 and 0.921, respectively, exceeding the threshold of good fit at 0.920. However, the CFI value of 0.743 fell below the desired value of at least 0.90.

In line with the direct effects model, both maternal height and alcohol consumption exhibited significant effects on FFGC, highlighting they are important factors on fetal growth. In addition, maternal BMI was found to have a significantly positive association with FFGC (β=0.12, 95% CI=0.03, 0.21). Furthermore, Hispanics positively affected FFGC compared to other race/ethnicity (β=0.14, 95% CI=0.02, 0.26). However, the influences of chemical exposure and food groups diverged from those of the direct effects model. In particular, DEHP showed a significant negative effect on FFGC (β=-0.16, 95% CI=-0.27, -0.04), while other chemical latent variables, DINOP, DM, DE, and BP did not yield significant effects. The diet latent variable along with total calorie intake, also manifested significant effects on FFGC. To be specific, the overall nutritional status represented by the diet latent variable demonstrated a positive effect (β=0.15, 95% CI=0.04, 0.25), while total calorie intake displayed a negative impact (β=-0.13, 95% CI=-0.22, -0.05) on FFGC.

The factor loadings for indicators of chemical exposure latent variables suggest a significant contribution of chemical metabolites to each latent variable. For latent variables DEHP, DM, and DE, all indicators exhibited factor loadings exceeding 0.6, signifying substantial contributions within their respective latent variables. Conversely, mCHpP, a metabolite of DINOP, displayed a slightly lower factor loading of 0.45, in comparison to other metabolites, mCPP and mCiOP, which surpassed the value of 0.6. On the other hand, indicators of the BP latent variable, BPA and BPS, had loadings of 0.50 and 0.48, respectively, which were relatively smaller compared to indicators of other latent variables. Moreover, the factor loadings of food groups for the diet latent variable indicated that meat, grains, and vegetables were the most influential nutrition as their loadings were the largest three, 0.71, 0.69, and 0.63, respectively. The factor loadings of the remaining indicators for the latent variables are provided in S5 Table.

## 4. Discussion

SEM significantly enhances our understanding of fetal development and maternal health by enabling the simultaneous exploration of the intricate relationships between maternal characteristics, chemical exposures, and dietary factors with various birth outcomes. By incorporating FFGC as a latent variable, SEM provides a more integrated perspective of how these factors influence fetal development. It also allows for the identification of direct and indirect pathways through which maternal factors, environmental exposures, and dietary intake impact birth outcomes, offering insights into their complicated relationships.

Our study provides valuable insights into the multifaceted determinants of fetal growth and development, highlighting the complex interplay of maternal characteristics, chemical exposures, and dietary factors. Our findings underscore the significance of considering a broad range of factors in assessing fetal growth conditions and birth outcomes. Maternal characteristics such as height, BMI, and ethnicity emerged as significant predictors of FFGC, indicating the pivotal role of maternal physiology and demographics in shaping fetal development. Additionally, lifestyle factors such as alcohol consumption exhibited notable associations with FFGC, emphasizing the importance of maternal behavior in influencing fetal growth trajectories. These findings emphasize the need for comprehensive prenatal care that addresses maternal health and lifestyle factors to promote optimal fetal growth and development.

Moreover, our analysis of chemical exposures and dietary factors uncovered nuanced relationships between FFGC and birth outcomes. While only DEHP exhibited a significant negative effect on FFGC among the chemical exposures considered, this highlights the importance of investigating specific chemicals’ impacts on fetal development. Our findings align with the previous studies that have reported negative effects of DEHP exposure on birth outcomes, including preterm delivery, decreased gestational age, and reduced birth weight [48–50]. Additionally, Ferguson et al. [51] identified two DEHP metabolites, MEHP and MECPP, as being associated with increased odds of preterm birth. Furthermore, our analysis of dietary factors revealed both positive and negative effects on FFGC, with overall nutritional status positively influencing fetal growth while total calorie intake had a negative impact. These findings underscore the complexity of nutritional influences on fetal development and suggest the importance of balanced nutrition during pregnancy to promote optimal birth outcomes.

We also observed potential confounding by demographic and dietary factors across race/ethnicity groups. Hispanic mothers in our study were significantly younger and had lower lifetime alcohol consumption than non-Hispanic mothers. Additionally, their dietary patterns differed, with a higher intake of fruit, dairy, meat, and beans/peas but lower intake of seafood, nuts/seeds, and soy products. These variations may have influenced birth outcomes and FFGC, contributing to the observed associations. The observed race/ethnicity differences emphasize the need for careful interpretation of findings in epidemiologic studies, as lifestyle and demographic factors can introduce unexpected confounding effects.

We observed clear differences between the results from the direct effects model and the latent variable model. These discrepancies may arise from the methodological differences inherent in each approach. The direct effects model focuses on the individual relationships between predictors and outcomes, often missing the intricate interconnections and mediating effects. In contrast, the latent variable model captures the underlying constructs that integrate multiple observed variables, offering a more flexible and comprehensive understanding of the phenomena. By incorporating FFGC as a latent variable, we were able to extract and represent key information from observed variables, providing deeper insights into the impacts of maternal characteristics, chemical exposures, and dietary factors on fetal development. This approach revealed indirect pathways that might otherwise be overlooked in traditional direct effects models. The use of SEM with latent variables thus proves essential in studying fetal growth, as it allows for a more nuanced perspective on the underlying mechanisms shaping development.

Several studies have employed an SEM to demonstrate the existence of a latent variable FFGC in diverse populations. Bollen et al. [26] investigated whether birth weight, birth length, and gestational age are distinct factors with distinct causes or if it is plausible to view them as having a common dependence on a latent FFGC variable. Camerota et al. [27] explored whether the FFGC latent variable model replicates Bollen et al. [26] and generalizes to a different country (US) and time period. Kihal-Talantikite et al. [28] replicated Bollen et al. [26]’s approach in a population of newborns in Paris and examined the potential differential effect of the FFGC latent variable according to the neighborhood socioeconomic level. Furthermore, Mitku et al. [29] studied the influence of a range of factors, including socio-demographic, behavioral, nutritional, physical activity, and environmental variables, on birth outcomes, through the mediation of FFGC. Moreover, Moyo et al. [30] explored how various factors contribute to FFGC in pregnant individuals living with and without human immunodeficiency virus in the South African population.

There are only a few studies that utilize SEM to examine the effect of chemical exposures and nutrition on birth outcomes. da Mota Santana et al. [52] investigated the association between maternal dietary patterns and infant birth weight in the Brazilian population, while Chen et al. [53] explored the same relationship in the Taiwanese population. Both studies utilized EFA to identify latent dietary patterns. Furthermore, Moyo et al. [30] studied the effects of nutritional factors on a latent variable FFGC. Mitku et al. [29] studied the effect of prenatal NOx exposure on FFGC, while Mitku et al. [54] investigated the effects of multiple air pollution exposures, PM2.5, NOx, and SO2 on various birth outcomes, low birth weight, small for gestational age, and preterm birth. In addition, Song et al. [55] identified the negative impact of prenatal phthalate exposure on birth weight in Chinese neonates.

While we have substantially extended previous studies exploring the association between chemical exposures and dietary factors with birth outcomes, there are several limitations in this study. First, our analysis was based on a selective sample of 538 individuals from the full cohort who had completed data on dietary assessments and mental health questionnaires. This selectivity may introduce bias, as dietary intake and mental health status within the broader cohort remain unknown. Another limitation is the lack of data on additional determinants of fetal growth (e.g., genetics [56] and physical activity [57,58]), which may further influence birth outcomes. Moreover, our use of the pregnancy average of the urine samples to assess chemical exposures may not capture longitudinal variations in exposure levels throughout pregnancy accurately. Finally, our analysis included only select classes of environmental chemical exposures, limiting the scope of the findings. Addressing these limitations and conducting further research with more diverse populations will be essential for advancing our understanding of the complex determinants of fetal growth and development.

## 5. Conclusions

Our study demonstrates the interplay of maternal characteristics, environmental chemical exposures, and dietary factors on fetal growth and birth outcomes using both traditional linear regression and SEM. With the concept of FFGC latent variable, SEM revealed the detrimental impact of specific chemical exposures such as DEHP and the complex effects of dietary intake on fetal growth, reinforcing the importance of balanced nutrition during pregnancy. The distinct results from the direct effect models and the latent variable models illustrate the value of employing SEM in understanding the multifaceted determinants of fetal growth, advocating for further research to enhance maternal and child health outcomes.

## Supporting information

S1 Fig Data flow diagram. (TIF)

S2 Table Summary of maternal characteristics of excluded subjects and comparison with the final dataset included in the analysis. (DOCX)

S3 Table Below Limit of Detection (LOD) proportion, Median and Interquartile ranges (IQR) for included bisphenol, phthalate, and OP metabolites. (DOCX)

S4 Fig Correlation plot of chemical exposures included in the analysis. (TIF)

S5 Table Factor loadings of indicators for latent variables from FFGC latent variable model. (DOCX)

## Abbreviations

SEM: Structural equation modeling
NYU CHES: New York University Children’s Health and Environment Study (NYU CHES)
FFGC: Favorable Fetal Growth Conditions
DEHP: Di(2-ethylhexyl) phthalate
CI: Confidence interval
OP: Organophosphate
BP: Bisphenol
DAP: Dialkyl phosphate
IQR: Interquartile range
DHQ-II: Diet History Questionnaire II
PHQ-9: Patient Health Questionnaire 9
DAG: Directed acyclic graph
EFA: Exploratory factor analysis
RMSEA: Root Mean Square Error of Approximation
SRMR: Standardized Root Mean Square Residual
CFI: Comparative Fit Index
SD: Standard deviation.
