# Supplementary figures and images for: Effects of chemical exposures and diet on birth outcomes in a New York City pregnancy cohort: Mediation through favorable fetal growth conditions
Source: PLoS One. 2025 May 28;20(5):e0322399. doi: 10.1371/journal.pone.0322399 (PMC12118982; doi:10.1371/journal.pone.0322399)

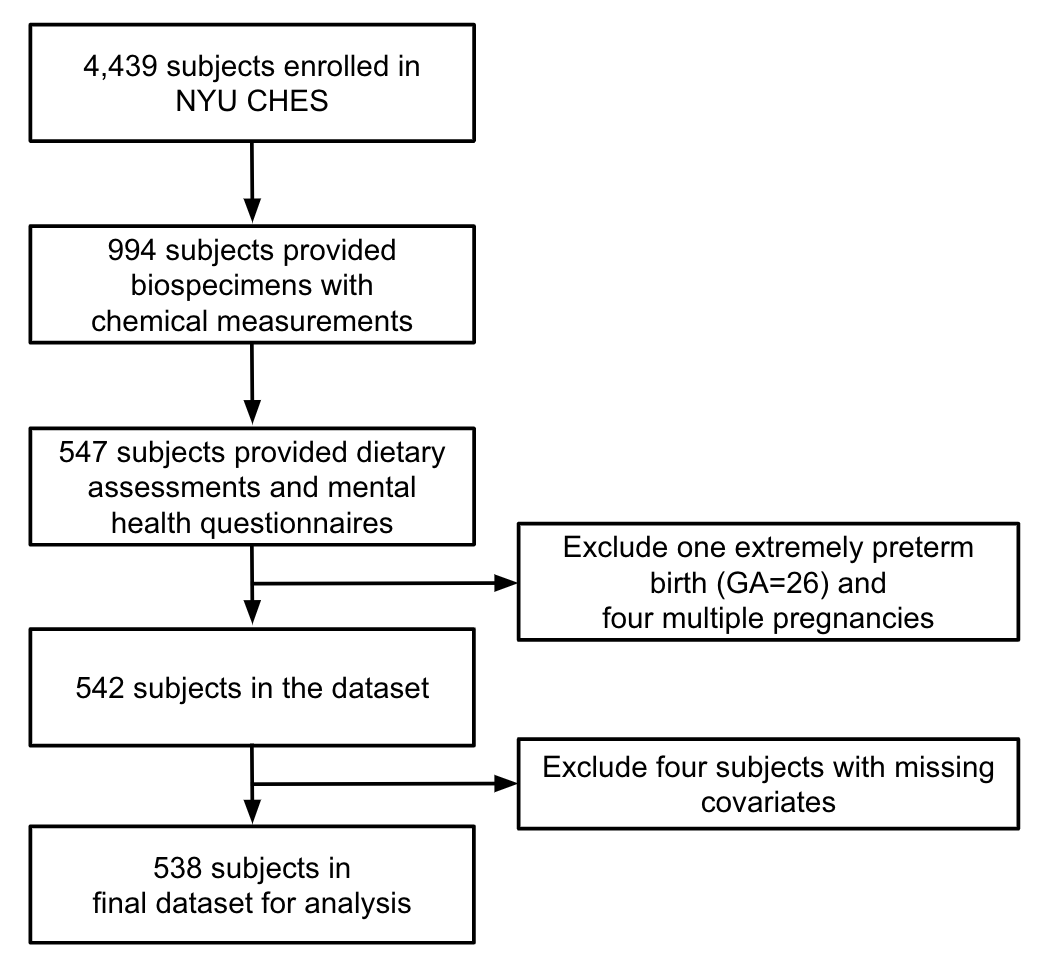

Supplement: S1 Fig — (TIF) [file pone.0322399.s001.tif]

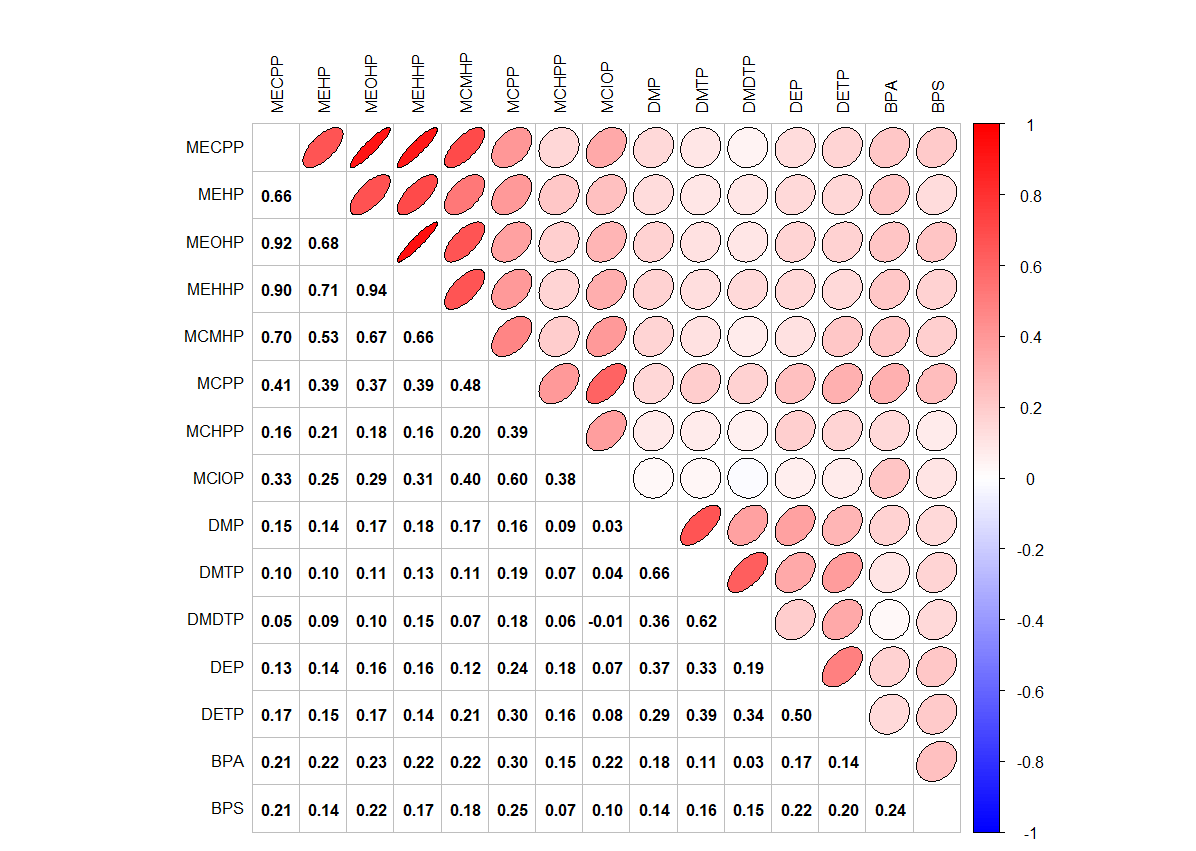

Supplement: S4 Fig — (TIF) [file pone.0322399.s004.tif]
