# Supplementary material for: Effects of chemical exposures and diet on birth outcomes in a New York City pregnancy cohort: Mediation through favorable fetal growth conditions
Source: PLoS One. 2025 May 28;20(5):e0322399. doi: 10.1371/journal.pone.0322399 (PMC12118982; doi:10.1371/journal.pone.0322399)
Supplement: S2 Table — (DOCX) [file pone.0322399.s002.docx]

**S2 Table: Summary of maternal characteristics of excluded subjects and comparison with the final dataset included in the analysis**

| **Variable** | **Remaining cohort**^a^  **(n=3901)** | **Final dataset**^b^  **(n=538)** | **p-value**^c^ |
| --- | --- | --- | --- |
| Age (years), mean (SD) | 32.19 (5.61) | 31.72 (5.53) | 0.07 |
| Height (cm), n^d^=3790, mean (SD) | 160.52 (8.51) | 160.82 (8.57) | 0.37 |
| BMI (kg/m^2^), n=3756, mean (SD) | 26.51 (5.87) | 25.95 (5.52) | 0.03 |
| Parity, n=3872, n (%) |  |  | 0.06 |
| Nulliparous | 1805 (46.62) | 275 (51.12) |  |
| Race/ethnicity, n=3762, n (%) |  |  | 0.36 |
| Hispanic | 1789 (47.55) | 256 (47.58) |  |
| Non-Hispanic White | 1300 (34.56) | 185 (34.39) |  |
| Non-Hispanic Black | 222 (5.90) | 28 (5.20) |  |
| Asian | 338 (8.98) | 50 (9.30) |  |
| Multiracial/Other | 113 (3.01) | 19 (3.53) |  |
| Education, n=3237, n (%) |  |  | 0.61 |
| High school or less | 1025 (31.67) | 167 (31.04) |  |
| Some college | 335 (10.35) | 59 (10.97) |  |
| Associate degree | 133 (4.11) | 30 (5.58) |  |
| Bachelor’s degree | 771 (23.82) | 125 (23.23) |  |
| Post-graduate degree | 973 (30.06) | 157 (29.18) |  |
| Employment status, n=3580, n (%) |  |  | 0.05 |
| Yes | 2301 (64.27) | 370 (68.77) |  |
| Alcohol intake (ever), n=3296, n (%) |  |  | 0.73 |
| Yes | 2191 (66.47) | 353 (65.61) |  |

^a^Dataset of 3901 subjects excluded from the analysis; ^b^Final dataset of 538 subjects used in the analysis; ^c^Wilcoxon rank sum test was used for age, height, and BMI, while the chi-square test was conducted for parity, race/ethnicity, education, employment status, and alcohol intake. Note that missing data were excluded from these comparisons. ^d^Sample size of the Remaining cohort for each variable, if any data are missing.
