# Supplementary material for: Effects of chemical exposures and diet on birth outcomes in a New York City pregnancy cohort: Mediation through favorable fetal growth conditions
Source: PLoS One. 2025 May 28;20(5):e0322399. doi: 10.1371/journal.pone.0322399 (PMC12118982; doi:10.1371/journal.pone.0322399)
Supplement: S3 Table — (DOCX) [file pone.0322399.s003.docx]

**S3 Table: Below Limit of Detection (LOD) proportion, Median and Interquartile ranges (IQR) for included bisphenol, phthalate, and OP metabolites**

| **Chemical**^a^ **or Chemical Group** | **% <LOD**^b^ | **Median (IQR)** |
| --- | --- | --- |
| Bisphenols (BP) | - |  |
| Bisphenol A (BPA) | 18.04 | 0.97 (1.20) |
| Bisphenol S (BPS) | 21.34 | 0.62 (0.87) |
| Phthalates | - |  |
| Di-(2-ethylhexyl) phthalate (DEHP) | - |  |
| Mono-(2-ethyl-5-carboxypentyl) phthalate (mECPP) | 0.56 | 7.11 (6.83) |
| Mono-(2-ethylhexyl) phthalate (mEHP) | 32.91 | 1.70 (2.60) |
| Mono-(2-ethyl-5-oxohexyl) phthalate (mEOHP) | 0.48 | 4.41 (4.06) |
| Mono-(2-ethyl-5-hydroxyhexyl) phthalate (mEHHP) | 0.44 | 6.98 (6.28) |
| Mono-(7-carboxyheptyl) phthalate (mCMHP) | 11.80 | 3.06 (3.47) |
| Di-n-octyl phthalate (DnOP) | - |  |
| Mono-(3-carboxypropyl) phthalate (mCPP) | 24.28 | 0.87 (1.07) |
| Mono-(7-carboxyheptyl) phthalate (mCHpP) | 51.11 | 0.50 (1.87) |
| Diisononyl phthalate (DiNP) | - |  |
| Mono-(carboxyisooctyl) phthalate (mCiOP) | 2.15 | 2.24 (3.09) |
| Organophosphate (OP) pesticides | - |  |
| Dimethyl phosphate metabolites (DM) | - |  |
| Dimethylphosphate (DMP) | 5.41 | 3.34 (3.82) |
| Dimethylthiophosphate (DMTP) | 0.72 | 2.93 (5.32) |
| Dimethyldithiophosphate (DMDTP) | 20.31 | 0.40 (1.24) |
| Diethyl phosphate metabolites (DE) | - |  |
| Diethylphosphate (DEP) | 2.66 | 4.01 (3.85) |
| Diethylthiophosphate(DETP) | 19.95 | 0.53 (0.85) |

^a^Units are ng/mL; ^b^Percentiles below the LOD are calculated as the ratio of the number of observations below the LOD to the total number of observations.
