# Supplementary material for: Effects of chemical exposures and diet on birth outcomes in a New York City pregnancy cohort: Mediation through favorable fetal growth conditions
Source: PLoS One. 2025 May 28;20(5):e0322399. doi: 10.1371/journal.pone.0322399 (PMC12118982; doi:10.1371/journal.pone.0322399)
Supplement: S5 Table — (DOCX) [file pone.0322399.s005.docx]

**S5 Table: Factor loadings of indicators for latent variables from FFGC latent variable model**

| **Latent variable** | **Indicator** | **Estimate** | **95% CI^a^** |
| --- | --- | --- | --- |
| DEHP | mECPP | 0.94 | (0.93, 0.95)* |
|  | mEHP | 0.72 | (0.67, 0.76)* |
|  | mEOHP | 0.97 | (0.96, 0.98)* |
|  | mEHHP | 0.96 | (0.95, 0.97)* |
|  | mCMHP | 0.71 | (0.66, 0.75)* |
| DINOP | mCPP | 0.91 | (0.85, 0.97)* |
|  | mCHpP | 0.45 | (0.38, 0.53)* |
|  | mCiOP | 0.66 | (0.60, 0.73)* |
| DM | DMP | 0.67 | (0.62, 0.73)* |
|  | DMTP | 0.98 | (0.94, 1.03)* |
|  | DMDTP | 0.63 | (0.57, 0.69)* |
| DE | DEP | 0.67 | (0.59, 0.74)* |
|  | DETP | 0.75 | (0.67, 0.82)* |
| BP | BPA | 0.50 | (0.39, 0.61)* |
|  | BPS | 0.48 | (0.37, 0.58)* |
| Diet | Vegetables | 0.63 | (0.57, 0.70)* |
|  | Fruit | 0.34 | (0.26, 0.43)* |
|  | Grains | 0.69 | (0.63, 0.74)* |
|  | Dairy | 0.49 | (0.42, 0.57)* |
|  | Meat | 0.71 | (0.66, 0.77)* |
|  | Seafood | 0.48 | (0.41, 0.56)* |
|  | Egg | 0.54 | (0.47, 0.61)* |
|  | Nuts & seed | 0.34 | (0.26, 0.43)* |
|  | Beans & peas | 0.45 | (0.37, 0.53)* |
|  | Soy products | 0.26 | (0.17, 0.35)* |

**^a^**CI: Confidence interval; *Significant at the significance level 𝛼=0.05
